# A missing piece in the Health for Peace agenda: gender diverse leadership and governance

**DOI:** 10.1136/bmjgh-2021-007742

**Published:** 2022-10-05

**Authors:** Kristen Meagher, Hala Mkhallalati, Nassim El Achi, Preeti Patel

**Affiliations:** 1 Department of War Studies, King's College London, London, UK; 2 Saw Swee Hock School of Public Health, National University Singapore, Singapore; 3 School of Geography and the Environment, Oxford University, Oxford, UK

**Keywords:** health systems

## Abstract

The purpose of this paper is to explore how gender diverse leadership and governance of health systems may contribute to the Health for Peace Agenda. Despite recent momentum, the evidence base to support, implement and evaluate ‘Health for Peace’ programmes remains limited and policy-makers in conflict settings do not consider peace when developing and implementing interventions and health policies. Through this analysis, we found that gender diverse leadership in health systems during active conflict offers greater prospects for sustainable peace and more equitable social economic recovery in the post-conflict period. Therefore, focusing on gender diversity of leadership and governance in health systems strengthening offers a novel way of linking peace and health, particularly in active conflict settings. While components of health systems are beginning to incorporate a gender lens, there remains significant room for improvement particularly in complex and protracted conflicts. Two case studies are explored, north-west Syria and Afghanistan, to highlight that an all-encompassing health systems focus may provide an opportunity for further understanding the link between gender, peace and health in active conflict and advocate for long-term investment in systems impacted by conflict. This approach may enable women and gender minorities to have a voice in the decision-making of health programmes and interventions that supports systems, and enables the community-led and context-specific knowledge and action required to address the root causes of inequalities and inequities in systems and societies.

**Summary box**

- Focusing on gender diversity of leadership and governance in health systems strengthening offers a novel way of linking peace and health, particularly in active conflict settings.
- Gender diverse leadership in health systems strengthening during active conflict offers greater prospects for sustainable peace and more equitable social economic recovery in the post-conflict period.
- Community-led and context-specific knowledge and action are required to address the root causes of inequalities and inequities in health systems strengthening in active conflict settings.
- Evidence to support, implement and evaluate ‘Health for Peace’ programmes remains sparse and does not holistically include wider discussions related to gender equity and equality.

## Background

Despite recent global momentum on ‘Health for Peace’ programmes, gender equality movements and development agendas, the evidence base to explore how these are interconnected, particularly in armed conflict, does not exist. Yet, public health interventions alongside social sector reform can contribute to peacebuilding in the aftermath of conﬂict and evidence suggests that the greater the level of gender inequality in a country, the higher the chances of conflict. Gender equality and equity movements and development agendas translate across sectors and systems and may, therefore, provide the impetus to reformulate the peace and health nexus.

In this paper, we explore how health systems strengthening is beginning to incorporate a gender lens, although there is significant room for improvement, particularly in complex and protracted conflicts.

While the current evidence is limited, public health measures, including equitable access to basic healthcare, may contribute to peacebuilding in the aftermath of conﬂict, so that health system itself can play a much wider role if we diversify thinking beyond traditional paradigms, for example, contributing to peacebuilding in conflict settings.^1^ Yet, this lacks significant empirical evidence. Given the global status of protracted conflicts and that most of the world’s extreme poor could live in fragile, conflict and violence-affected settings by 2030, and the exacerbation of this through COVID-19, investment in research and understanding how to advance health systems for peace has never been more critical.^2 3^

Two of the major active and continuously evolving conflicts, north-west Syria and Afghanistan, will be used herein to highlight that an all-encompassing health systems focus may provide an opportunity for further exploring the link between gender, peace and health in active conflict and advocate for long-term investment in systems impacted by conflict. Furthermore, this may support the high-level, international policy recommendations from bodies such as the UN and the World Bank to have much more policy relevance, rigour and impact at the local level.

## Health systems strengthening and peace

Despite the increasing plethora of research on health systems across objectives, functional and organisational arrangements, there is a lack of consensus on what constitutes health systems strengthening.^4^ The WHO defines health systems strengthening as ‘any array of initiatives that improves one or more of the functions of the health systems and that leads to better health through improvements in access, coverage, quality or efficiency’.^5^ These functions, as defined by the WHO Health Systems Framework 2000 and the six major Building Blocks include: service delivery, health workforce, health information systems, access to essential medicines, financing and leadership/governance.^5^ It was envisioned that the Building Blocks could be used to assess and strengthen health systems; however, some authors argued that health outcomes are the best indicators of a health system’s performance.^6^ While many others consider that health system strengthening is a means to achieve Universal Health Coverage which embodies a set of criteria, including quality, equity, efficiency, accountability, resilience and sustainability, that are in accordance with the priority areas of action of the Sustainable Development Goals (SDGs) 2030.^7 8^ We aim to explore leadership and governance from a gendered aspect in conflict settings.

In reviewing health systems strengthening frameworks and definitions, it is evident that these may not be suitable to measure and understand the significance of the role of gender in health systems strengthening in contexts affected by active conflict. Gender is embedded within health systems, is part of the broader social determinants of health, is important in shaping health outcomes, but is external to the health system.^9^ Thus, most established models and frameworks representing health systems do not incorporate gender as a core component, thereby reinforcing often restrictive gender norms and gender inequities and inequalities within health and the system more broadly. Such frameworks need to go further to understand the relationship between various factors, including biomedical approaches, the socioeconomic determinants of health and the social construction of gender, and its direct impacts on structural gender inequalities.^10 11^

Analysis from van Olmen *et al* states that the transformation of health systems thinking over time does not reflect a progressive accumulation of insights and lessons learnt.^12^ Instead, theories and frameworks have developed in reaction to one another and are not neutral, framing health, health systems and policies within political and public health paradigms.^12^ Contrasting examples include the reform perspective, which considers health systems as ‘projects to be engineered’, the organic view that health systems are a mirror of society and the co-existence of health systems and disease centric, vertical programmes.^12–14^

Similarly, health systems governance incorporating strategic vision, participation, transparency, responsiveness, equity, effectiveness, accountability and information, as core elements, has multiple definitions and frameworks with no single, agreed framework that can serve all purposes of governance and its contribution to health systems strengthening. This results in challenges in having governance as an actionable health system function. A governance triangle listed in the 2004 World Development Report, and further adapted by multiple scholars to provide additional frameworks, reflects how governance is to be achieved in practice by exploring key (complex) relationships between and within three categories of stakeholders: policy-makers, providers of health services and people. With the latter being the diverse users of health services, little is mentioned specifically in the literature about gender equity and equality in governance.^15 16^ Moreover, the impact of context on health system governance is not well researched and needs to be further acknowledged and explored.

The concept of peace through health has a long history. The WHO envisages health as a neutral starting point in convening the myriad of actors in conflict, as they work towards shared goals.^17^ In 1998, the WHO adopted ‘Health as a Bridge for Peace’ as a policy framework with the premise that the role of healthcare workers would extend to the preservation and promotion of peace.^18^ While the WHO programme has ebbed, partly due to the challenges of proving a ‘tangible peace dividend’, active research has continued ‘peace-through-health’ mechanisms.^19^ Despite more recent momentum, the evidence base to support, implement and evaluate ‘Health for Peace’ programmes remains limited and policy-makers in conflict settings do not consider peace when developing and implementing interventions and health policies.^2^ To further drive the research agenda, developed peacebuilding mechanisms may support this. In 1997, Lederach developed a peacebuilding infrastructure, which, along with other objectives, sought to understand the dynamic interplay and interdependence between the various levels of society affecting change processes.^20^ Therefore, further exploration of peacebuilding mechanisms, and how these mechanisms can translate into health systems strengthening that incorporate a gender lens at the leadership and decision-making level, may be an innovative avenue to enhance understanding of how health and peace interact.

Criticisms persist that peace through health efforts reflects ideology rather than evidence and that the altruistic basis for health action fundamentally contradicts interest-based peace negotiations.^18^ In Bosnia-Herzegovina, the health system, like many other systems, was divided between the Bosnian Croats and Bosnian Serbs. WHO and the UK’s Department for International Development collaborated to unify staffing, service provision and delivery of healthcare, which they claim, reduced separatist attitudes.^21^ Immunisation drives targeting whole populations can provide entry point opportunities for education and outreach or ‘peace messaging’, as used across Central America and Sudan.^22^ Critics, however, point to instances where health programmes were designated with political or strategic interests in mind, such as US involvement in Afghanistan mostly through Provincial Reconstruction Teams, to bolster military forces and prolong fighting, or food aid delivery to the Sudanese military in 1998, resulting in a piecemeal system of non-governmental organisation (NGO) or private delivery, exacerbating inequities and fuelling conflict.^18 23^ While these criticisms hold validity and must be reflected on when developing and implementing health interventions in conflict settings, they do not consider the role of gender at a health systems level as an avenue for supporting peace through health in conflict.

## Bridging health and peace through gender diverse leadership and governance

Globally, there is strong momentum for advancing gender equity and equality embedded in feminist movements and development agendas, which also emphasises their interconnectedness. These include the SDGs: SDG 3, promoting good health and well-being; SDG 5, advancing gender equality; SDG 10, reducing inequality within and among countries; SDG 16, promoting peaceful and inclusive societies; United Nations Security Council (UNSC) Resolution 1325 (2000), recognising women’s inclusion in peace and security; UNSC Resolution 2493 (2019), calling for the implementation of all prior resolutions on women, peace and security; Gender Equality Forum in July 2021 and the Global Acceleration Plan to Advance Gender Equality by 2026; MeToo movement; and Time’s Up.^24–29^ These movements and agendas translate across sectors and systems and may provide the much-needed impetus to reformulate the peace and health nexus. Health and peace through a gender lens offer novel ways of thinking by incorporate different points of view, and theories to be explored that support sustainable peacebuilding. Percival *et al* states that the gender-blind nature of health system engagement has missed an important opportunity to contribute to more equitable and peaceful societies, given the frequent contact made by individuals with health services, and the important role of the health system within societies.^30^

Conflict promotes conditions during which existing gender inequalities and inequities are amplified. It is estimated that women and children make up around 80% of internally displaced populations, women of reproductive ages living near high intensity conflicts have three times higher mortality than do women in peaceful settings and in 2020 43% of civilians killed in Afghanistan were women and children.^31–33^ It is well evidenced that women’s participation in economic and political life is vital for conflict prevention and resolution.^34^ Moreover, women’s participation in peace negotiations with voice and influence leads to better accord content, higher agreement implementation rates and longer lasting peace.^35^ Krause *et al*’s research assumes that the inclusion of women in leadership positions may be more likely to allow for women’s political participation and generally support norms of gender equality, which can have an independent effect on the durability of peace.^35^ Kruk *et al*’s study argues that rebuilding health services can play an essential role in promoting social cohesion in a nation’s post-conflict recovery stage; yet, supportive empirical evidence is thin.^36^ Our recent study shows that women’s inclusion in the political economy of health in conflict has greater dividends for sustainable peace and more equitable social economic recovery in the post-conflict period.^37^ Yet, the conflict literature features very limited discussions on the important role of gender equity and equality within a broader health systems emphasis; its focus to date has been predominantly in very few post-conflict settings.

There is widespread consensus among practitioners and scholars that peacebuilding can be more effective if built on an understanding of how gendered identities are constructed through societal power relations between and among women, men, girls, boys and members of sexual/gender minorities.^38 39^ Findings from a study in Uganda linking education, gender and peace demonstrate gender equality and sustainability in peacebuilding through public institutions and social services cannot be detached from how rigid gender roles and persistent power dynamics are culturally, socially, politically and economically perpetuated and reproduced.^38^ Growing evidence suggests that the greater the level of gender inequality in a country, the higher the chances of conflict.^38^ Building on this, strengthening health systems in conflict settings through a gender lens and in particular, at a leadership level, may support dismantling the entrenched practice of creating gender-blind social institutions. Incorporating women in the earliest phases of health sector rehabilitation has been identified as crucial, as they have likely been marginalised from public life, yet are often the eyes and ears of families and communities. In countries like Afghanistan, the healthcare system has discriminated against women, both in terms of their role as healthcare professionals and service delivery. Including women in policy discussions and human resource development will provide more opportunities to ensure a distribution of resources that will be accessible to, and used by, women and their children.^40^ This, however, needs to be prioritised at the community and national level, not simply through international NGOs and donors.

Taking a health systems approach reinforces the value of incorporating gender as an essential component of health in conflict; women are disadvantaged by the structures that influence health systems in conflict and are frequently excluded from decision-making in not only health, but broader systems during active conflict; they are also disproportionately impacted by armed conflict. Therefore, cultivating and harnessing the advancements of women’s meaningful leadership, that includes decision-making, at community, national and international levels, and acknowledging the significance of their contribution to health systems strengthening in conflict and humanitarian crises is paramount.^41^ This will in turn create effective, community-led and, therefore, contextually appropriate leadership models, that influence decision-making in health systems that may in turn contribute to sustainable peace building.^41^

## Case studies

### Afghanistan

Since the initial fall of the Taliban in 2001, Afghanistan made considerable gains in health despite the protracted conflict affecting many areas of the country.^42^ There has been substantial improvements in maternal and child mortality, yet broader gender gaps remain.^43^ The establishment of a Basic Package of Health Services (BPHS) in 2003, and Essential Package of Hospital Services in 2005, as foundations for an equitable healthcare system has contributed towards improving many health indicators and improving leadership for women in the health sector.^44^ The implementation of the BPHS was achieved through a contracting mechanism between donors, the Ministry of Public Health and NGOs, and represented one of the largest investments in health in a conflict-affected country. Before the BPHS, Afghanistan had the second highest maternal mortality in the world.^45^ There were wider gains too. Afghan women fought for their own rights and took a proactive role in the development of human rights in their nation, including the establishment of Afghanistan Independent Human Rights Commission.^46^ There was a Ministry of Women’s Affairs and, in 2009, a landmark law was passed to address violence against women. Afghanistan has also become a signatory to several international human rights instruments, such as the Convention on the Elimination of All Forms of Discrimination Against Women.^46^ In 2020, about one-fifth of Afghan civil servants were women and one in four parliamentary seats were held by women—up from 0 in 2001.^47^

Studies of the Afghanistan experience show that while improving health indicators for women, the BPHS did not sufficiently reflect on if and how to promote gender equity within the health system: female health workers and the provision of primary healthcare services was a tool to reduce maternal mortality—not part of the effort to build a gender equitable health system or promote gender equity.^9^

The future remains uncertain for women and girls under the Taliban. There are fears that female health workers will not be allowed to work, and that women will be prevented from pursuing education, including those wishing to become doctors and nurses—hampering efforts in the last two decades to address shortages of female health workers, including midwives.^48^ In the southern city of Kandahar, women’s healthcare clinics have closed down and in some districts, girls’ schools have been closed since the Taliban seized control of them in November 2020.^47^ If an attempt to improve the understanding and thereby the value of the peace through health framework with gender equity and equality in leadership and governance at its core, there may have been an opportunity to strengthen not only the health system, but create a more peaceful and less patriarchal society, rather than the more narrow focus of only improving health outcomes.

### North-west Syria

The case of north-west Syria provides an array of challenges to health systems strengthening, and, furthermore, the inclusion of gender as a core component in strengthening such systems and progressing peacebuilding. Challenges include the weaponisation of healthcare and aid restrictions, which hinder health responses and enhance inequity in access to healthcare.^49 50^ Given the lack of research on how gender as a core component of building sustainable health systems, wider avenues for peacebuilding through health systems are dismissed.

While gender equality was not a primary focus of the Arab Spring and Syrian conflict, women took leading roles in mobilising the non-violent movement of the Syrian uprising. Despite their immense efforts, women’s participation in public, social and political life remains a contentious issue.^51^ Syria’s social structures, both prior to and since the emergence of conflict, are predominantly based on conventional gender roles and typically patriarchal, though they differ according to disparate cultural, social specificities and value systems across the country. This structure has been replicated across the various health systems in Syria. Women have contributed significantly to the health system throughout the conflict but remain invisible in leadership roles. This is largely because of a complex political system with an even more complicated and fragmented health system, a lack of support from colleagues and restrictive norms to women’s leadership.^51^ Rather than weaponising health systems and neglecting the role of gender diverse leadership, there may be an opportunity here for advancing peace through health systems in conflict settings.

Health systems and services in post-conflict settings are usually provided by humanitarian actors that tend to create parallel systems for provision of healthcare rather than supporting existing systems.^52^ As a result, it is very common to see emergent health systems collapse in the post-conflict phase.^53 54^ In the case of north-west Syria, there has been a sustained effort to rebuild the health system during the active conflict phase. The health system in the north-west was created using a bottom-up approach, connecting local medical bodies with a central core team in each governorate.^55^ To make best use of the limited resources and, in an effort to protect medical volunteer efforts from fragmentation, local Syrian healthcare workers formed ‘Syrian Health Directorates’ to coordinate aid and govern the health needs of the population.^55^ Indeed, a recent study focusing on rebuilding health system governance in opposition-controlled areas in Syria has shown that it is ongoing despite the conflict. However, the authors highlighted that political recognition of local health authorities and their financial and technical support are needed to support this process. More importantly, more pressure should be done by the international community to prevent the weaponisation of healthcare facilities and workers.^56^ How an explicit gender focus contributes to health system strengthening during the Syrian conflict like any other conflict has, however, been largely absent from evidence to date.

## Conclusion

The north-west Syria and Afghanistan contexts support the need to further explore the peace through health agenda by reorientating attention toward gender diverse leadership and governance within health systems, thereby going beyond direct health outcomes to focus on the systems themselves. This approach may enable women and gender minorities to have a voice in the decision-making of health programmes and interventions that subsequently feeds back into systems, thereby enabling the community-led and context-specific knowledge and action required to address the root causes of inequalities and inequities in systems and societies. Peacebuilding cannot be measured on statistical analysis alone, but how systems enable all individuals to have voice and participate meaningfully within societies.

Understanding health and peace through a gender lens that uses context-specific case studies and empirical evidence is, therefore, imperative. The nascent research to date demonstrates that by not including gender analysis in the peace and health nexus, there will remain significant setbacks to achieving sustainable peace. The development of a new research agenda through gathering empirical evidence in conflict settings is, therefore, required to, first, understand how profound the gender gap is; second, understand how effective including women or individuals of gender minorities equally in leadership and governance in health systems to support sustainable peace building or sustainable efforts is; and, third, to do so in a way that is community led from the onset to ensure gender inequity and inequality in positions of leadership is recognised as problematic at a grassroots level.

## Data Availability

All data relevant to the study are included in the article.
